# PD-1 mRNA expression in peripheral blood cells and its modulation characteristics in cancer patients

**DOI:** 10.18632/oncotarget.15006

**Published:** 2017-02-02

**Authors:** Wei Wang, Ge Shen, Shikai Wu, Shiping Song, Yanli Ni, Zhuoyao Suo, Xiangying Meng, Dan Li, Lin Zhou, Rimin Hao, Yaowei Zhao, Li Bai, Lili Hou, Bing Liu, Guangxian Liu

**Affiliations:** ^1^ Cancer Therapy Center, Affiliated Hospital of The Academy of Military Medical Sciences, Beijing 100071, China

**Keywords:** PD-1, peripheral immune cells, immunomodulation, radiation therapy, cancer

## Abstract

Immune checkpoint inhibitors that block the PD-1/PD-L1 signaling pathway have been used to treat a wide variety of cancers. Although results have been promising, significant inter-individual and inter-tumor variability has been observed. It is believed that better clinical outcome could be achieved if the treatment was individually designed based on the functional status of the PD-1/PD-L1 signaling and the cellular immunity. In this study, we analyzed the mRNA expression of PD-1 and other immunomodulatory genes in peripheral blood from cancer patients, and immunomodulatory gene expression during radiotherapy and immunomodulation therapy with cytokines. Our results show that the PD-1 mRNA expression is significantly increased in peripheral blood in cancer patients. Anti-cancer treatments can significantly modulate the PD-1 expression, but this is largely dependent on the initial immune status. Moreover, the PD-1 expression on peripheral lymphocytes can be immunoactivation-derived. These results suggest that the regulation and expression pattern of PD-1/PD-L1 signal is complicated which will influence the effect of blockade of the PD-1/PD-L1 signaling pathway for cancer treatment. Through combined analysis of PD-1, CTLA-4, and other immune markers in peripheral blood, we may accurately evaluate the functional status of PD-1/PD-L1 signaling and cellular immunity, thereby providing clues for guiding anti-PD-1 or anti-PD-L1 treatment.

## INTRODUCTION

Programmed death-1(PD-1,CD279), a member of the immunoglobulin superfamily, is an immune checkpoint receptor that is expressed on the surface of peripheral T cells, B cells, natural killer T (NKT) cells, dendritic cells(DC), and some monocytes. Upon binding to its ligands, programmed death-1 receptor ligand-1 (PD-L1) or -2 (PD-L2), the PD-1 engagement leads to inhibition of cell growth and cytokine secretion, normally serving as a feedback inhibitory mechanism of the immune system [1–6]. However, this mechanism has been linked to immune tolerance and therefore provides a possible mechanism of escaping immune surveillance when tumor cells become capable of expressing PD-L1 [7, 8].

Recently, blockade of the immune checkpoints using monoclonal antibodies against cytotoxic T-lymphocyte antigen-4 (CTLA-4), PD-1, and PD-L1, has shown striking clinical results in cancer patients [9–13]. Pembrolizumab is the first monoclonal antibody targeting PD-1 [11], which has been used in advanced melanoma patients [12, 14]. Nivolumab is another fully humanized monoclonal antibody against PD-1; it has been used in clinical trials to treat melanoma, non-small cell lung cancer, and renal cell carcinoma [15]. Although preliminary results from these clinical trials have been striking, they have not yet met the expectation for the potency of the new treatment strategy. In addition, a significant inter-individual and inter-tumor variability in response to these antibodies has been observed [16].

It is believed that better clinical outcomes could be achieved if the treatment is individually designed based on the functional status of the PD-1/PD-L1 signaling and the cellular immunity [17–18]. Early studies suggested that measurement of PD-1 on tumor infiltrated lymphocytes (TILs) and PD-L1 expression on tumor cells after surgery or biopsy might be advisable [19–22]. However, later studies indicated that PD-1 expression on peripheral blood leukocytes may provide a more useful and practical indicator of cancer progression, and may assist in identifying patients likely to respond to PD-1/PD-L1 blockade [23, 24]. However, since the relationship between the clinical response to PD-1 antibodies and the immune status is complicated, monitoring PD-1 expression on tumor infiltrated lymphocytes as well as PD-L1 on tumor cells is clinically impractical and insufficient for most patients. A more reliable and practical protocol for evaluating the functional characteristics of the PD-1 signaling pathway and the cellular immunity is urgently needed.

In this study, we have analyzed PD-1 mRNA expression in peripheral blood from cancer patients, and evaluated its modulation during clinical treatments with radiotherapy and immunomodulation therapy with cytokines. Our results show that PD-1 mRNA expression is increased in peripheral blood from cancer patients, and can be modulated by clinical procedures. Moreover, the PD-1 expression on peripheral lymphocytes can be immunoactivation-derived. Our data indicate that through combined analysis of PD-1, CTLA-4, CD25, CD28, Foxp3, TGF-β and IL-10 expression and the lymphocyte subpopulations in peripheral blood, we could accurately evaluate the functional status of cellular immunity and PD-1/PD-L1 signaling pathway, which could provide clues for guiding cancer treatment using anti-PD-1 antibodies.

## RESULTS

### PD-1 and CTLA-4 mRNA expression in healthy adults and cancer patients

**Figure 1:**
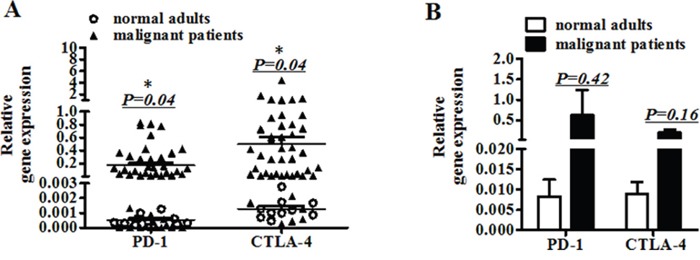
The gene expression of PD-1 and CTLA-4 in peripheral blood **A.** The mRNA expression of PD-1 and CTLA-4 in peripheral blood from ten normal adults and 45 patients. **B.** The mRNA expression of PD-1 and CTLA-4 in mononuclear cells (PBMC) separated from peripheral blood of normal adults and patients. (A) Significance of the data were calculated and denoted as *, p<0.05. (B) The data is representative of two independent experiments and have not significance.

Ten healthy adults and 45 cancer patients were analyzed for gene expression of PD-1, CTLA-4, CD25, CD28, IL-10, TGF-β and Foxp3 in peripheral blood. As shown in Figure 1A and Supplementary Tables 1 and 2, expression of PD-1 and CTLA-4 in cancer patients was significantly increased compared to that in healthy adults(p=0.04). Specifically, expression of PD-1 in 71% (32/45) and CTLA-4 in 87% (39/45) cancer patients was higher than in all ten healthy adults. Next, we analyzed PD-1 and CTLA-4 mRNA levels in mononuclear cells (PBMC) isolated from peripheral blood. As shown in Figure 1B, even though the gene expression of PD-1 and CTLA-4 in PBMC of cancer patients was increased, it did not reach a statistical significance; this will need to be further investigated with a larger cohort.

### Modulation of PD-1 and CTLA-4 mRNA expression during cancer treatment

Cellular immunity of 45 cancer patients, including 25 cases received immunomodulation therapy and 20 cases received radiotherapy, was monitored by flow cytometry. The proportion of T cells (70.05-71.29%), B cells (10.21-7.53%), NK cells(19.85-20.64%), Treg cells(3.32-3.74%), CD3^+^CD8^+^CD28^-^ cells(14.32-15.57%), and CD3^+^CD8^+^CD28^+^ cells(15.18-15.26%) showed no significant differences between before and after treatment (Supplementary Table 3).

**Figure 2:**
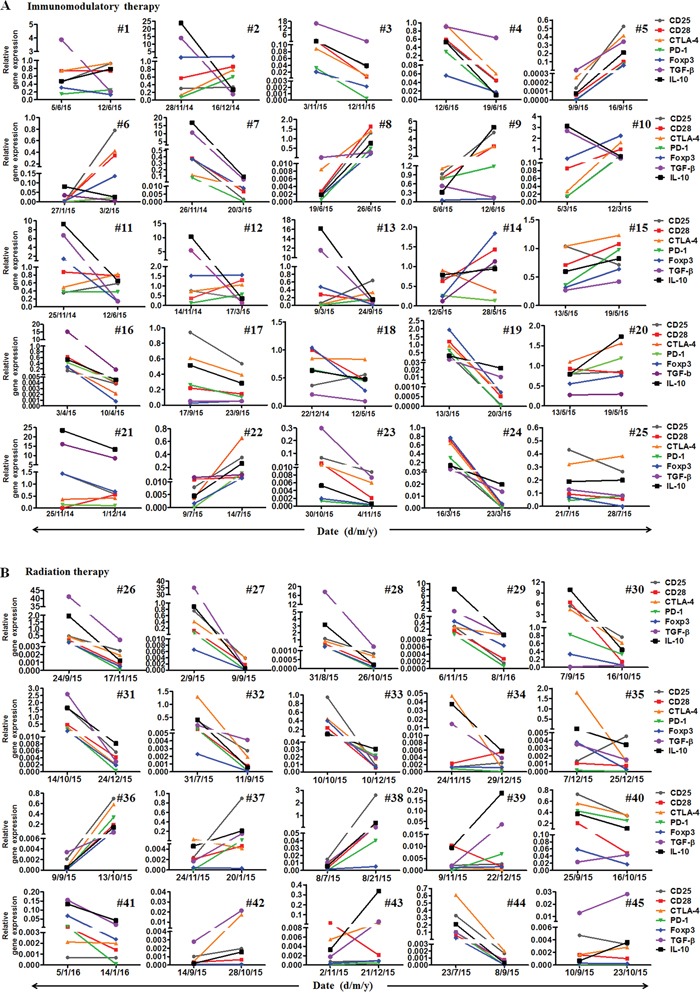
Monitoring mRNA expressions of seven immunoregulatory molecules including CD25, CD28, CTLA-4, PD-1, Foxp3, TGF-β and IL-10 **A.** The mRNA expression of the seven molecules in 25 malignant patients before and after immunomodulation therapy. **B**. The expression of the seven molecules in 20 malignant patients before, after and one month (IMRT) or two months (CyberKnife) after radiotherapy.

Interestingly, the PD-1 mRNA expression declined in 12 patients (48%) and increased in 13(52%) patients in the cohort with immunomodulation therapy. CTLA-4 mRNA expression decreased in 9 patients (36%) and increased in 16 patients (64%)(Figure 2A). The up- and down-modulation of PD-1 and CTLA-4 mRNA expression in the patients largely depended on the initial mRNA levels. If the initial expression was increased, it would be decreased after therapy, and vice versa.

**Figure 3:**
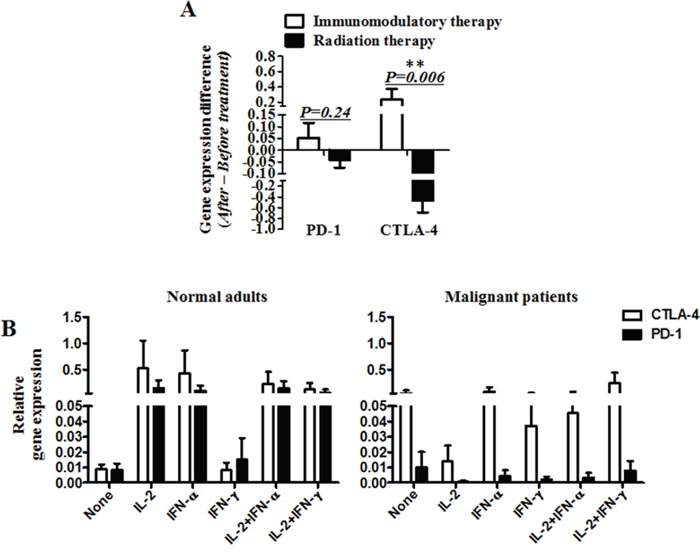
The mRNA levels of PD-1 and CTLA-4 *in vivo* and *in vitro* **A.** The mRNA levels of PD-1 and CTLA-4 after treatment minus that before for each patient. **B.** The mRNA expression of PD-1 and CTLA-4 in peripheral blood mononuclear cells (PBMC) from normal adults or malignant patients, stimulated by cytokines (IL-2, IFN-α and IFN-ɤ) or cytokine combination (IL-2+IFN- α and IL-2+IFN-ɤ). (A) Significance of the data were calculated and denoted as **, p<0.01. (B) The data is representative of two independent experiments and have not significance.

In the cohort with radiotherapy, PD-1 mRNA expression declined in 15 patients (75%), and increased in 5(25%) patients. CTLA-4 mRNA levels decreased in 15 patients (75%) and increased in 5 patients (25%)(Figure 2B). The up- and down-modulation of PD-1 and CTLA-4 mRNA expression in the patients also largely depended on the initial expression levels. Notably, there was a significant difference in the mRNA levels of PD-1 and CTLA-4 between radiotherapy and immunomodulation therapy. When the mRNA levels of PD-1 and CTLA-4 after treatment minus that before for each patient, it was found that the radiation cohort had much decreased mRNA levels of PD-1 and CTLA-4 than that in the immunomodulation cohort, with significant difference for CTLA-4 (p=0.006) but not for PD-1(p=0.24), see Figure 3A. Comparison of the influence of cytokines or cytokine combination on the PD-1 and CTLA-4 mRNA levels in PBMC from normal adults and cancer patients revealed that the PD-1 and CTLA-4 expression increased in most cancer patients prior to cytokine treatment, and was hardly modulated after the treatment (Figure 3B), indicating the intrinsic inhibition of the immune function by tumors. As there were several types of cancer in the immunomodulation cohort, we also compared the PD-1 and CTLA-4 mRNA expression between the 7 lung cancer patients and 18 other malignancies. The results showed that there was no significant difference between the two groups in PD-1 and CTLA-4 mRNA expression as well as in lymphocyte counts (data not shown).

### Correlation of PD-1 mRNA expression with other immunosuppressive genes

**Figure 4:**
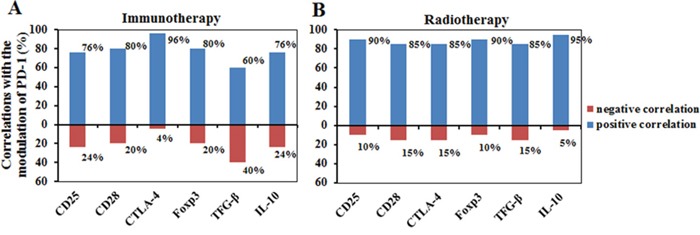
The correlation of PD-1 with six other functional immune molecules including CD25, CD28, CTLA-4, Foxp3, TGF-β and IL-10 after immunomodulation therapy A. or radiotherapy B Blue column, positive correlation; Red columns, negative correlation.

We have also measured mRNA levels of CD25, CD28, Foxp3, TGF-β and IL-10 during the treatments (Figure 2). The modulation of these genes by the treatments also largely depended on their initial levels. Interestingly, much larger bidirectional regulatory effect among these genes was found in the immunomodulation therapy cohort than in the radiation cohort. Particularly, in the cohort with immunomodulation therapy, PD-1 mRNA expression highly correlated with CTLA-4 (96%), followed by CD28 (80%), Foxp3 (80%), CD25 (76%) and IL-10 (76%), and negatively correlated with TGF-β (40%), CD25 (24%), IL-10 (24%), CD28 (20%), and Foxp3 (20%) (Figure 4A). However, in the cohort with radiation therapy, there was no significant reverse correlation among these signals. Instead, PD-1 mRNA expression correlated with CTLA-4 (85%), CD25 (90%), CD28 (85%), Foxp3 (90%), TGF-β (85%) and IL-10 (95%) expression, and reversely correlated with all the other molecules <15% (Figure 4B). These results revealed that the modulation mechanism of these functional molecules was different between the two kinds of therapy.

The expression of IFN-ɤ in peripheral blood was also monitored, but was undetectable by the RT-PCR method. In substitution, we monitored IFN-ɤ expression in blood CD4 and CD8 cells by flow cytometry. We found that CD3^+^CD4^+^IFN-ɤ^+^ and CD3^+^CD8^+^IFN-ɤ^+^ subpopulations were frequently modulated during the treatments, but there was no significant correlation between IFN-ɤ^+^ CD4 or IFN-ɤ^+^CD8 cell count and peripheral PD-1 expression(data not shown). Further investigation is needed with a large cohort.

## DISCUSSION

In the present study, we have analyzed PD-1 mRNA expression in peripheral blood leukocytes as well as mRNA expression of several key immunoregulatory genes before and after cancer treatments. Gene expression of PD-1 as well as CTLA-4, CD25, CD28, Foxp3, TGF-β and IL-10 was increased in most cancer patients compared to that in healthy adults (see Figure 1, Supplementary Tables 1 and 2). No significant difference in PD-1 and CTLA-4 mRNA expression and in lymphocyte counts were observed between 7 lung cancer patients and 18 other malignancies in the immunomodulation therapy cohort, suggesting dysregulated cellular immunity in cancer patients. *In vitro* data showed that these signals in peripheral immune cells from patients were hardly modulated in 24 hours by cytokines or their combination, with minimal fluctuation of PD-1 and the other signals, obviously different from that in normal immune cells, indicating functional abnormality or blockage of the immune cells.

Expression of PD-1, CTLA-4, CD25, CD28, Foxp3, TGF-β and IL-10 could be easily modulated with a broad range of clinical therapeutic procedures. Previous studies have shown that some treatments, such as hypomethylating agents (HMAs), 5-azacytidine and 5-aza-2′ deoxycytidine, can induce immune reaction alterations [25, 26]. Yang et al reported that, in a cohort of patients with MDS, CMML, AML treated with epigenetic therapy, PD-L1, PD-L2, PD-1 and CTLA4 expression was up-regulated and patients resistant to therapy had higher PD-1 and CTLA4 expression compared to patients who responded to therapy. Treatment of leukemia cells with decitabine resulted in a dose dependent up-regulation of the above genes. It is believed that many treatments or drugs can affect functional status of these signals, with special significance in cancer immunotherapy [27]. In this study, we observed a rapid modulation of PD-1 mRNA expression after radiation and immunomodulation therapy. Interestingly, the response of PD-1 mRNA expression upon clinical treatment was observed in each of the clinical interventions and largely depended on the initial expression level. In contrast, radiation therapy had much larger amplitude of the response. The same phenomenon was also observed in the responses of CD25, CD28, CTLA-4, TGF-β and IL-10, which seems to be derived from both immunoactivation and stimulation by the two kinds of therapy, as either of the two actions could not entirely explain the phenomenon and the data (see Figure 2). Further dynamic observation showed that the PD-1 mRNA expression by RT-PCR, the PD-1 expression on lymphocytes by cytometry and the IFN-ɤ expression in CD4 and CD8 by cytometry were not synchronous (data not shown), which could be influenced by their different expression time spans.

To date, most studies have focused on PD-1 expression on TILs and PD-L1 expression on tumor cells. The results have shown that upregulation of PD-1 on TILs or PD-L1 on tumor cells was associated with immunotolerance, exhaustion of T cells, and poor prognosis [28–34]. Several studies have found that persistent expression of PD-1 by T cells is highly indicative of an exhausted phenotype noted by a decrease in effector function and linked to poor prognosis and tumor recurrence [35–37]. Data from this study showed that PD-1 and CTLA-4 up-regulation in the cohort of immunomodulation therapy had positive correlation with CD25 and CD28, but negative correlation with TGF-β, and no correlation with Foxp3. These results indicate that the increased expression of PD-1 upon treatment could be caused by immunoactivation, different from that in TILs, which largely represent the PD-1 up-modulation induced by tumor, as reported previously [38–45].

The most impressive result was the correlation of PD-1 mRNA expression with that of other immunoregulatory molecules and the difference in immunomodulation during different therapies. Increased PD-1 expression could be cancer-derived [30–36] or immunoactivation-derived, but not specific. We found that the increased PD-1 mRNA expression was companied by modulation of other immune molecules, but with different patterns among individuals or treatments (Figure 4). Interestingly, there was positive or negative correlation between expression of these molecules in the cohort of immunomodulation therapy, but only minimal negative correlation could be found in the cohort of radiotherapy, with special interests coming from the modulation of TGF-β and IL-10. In the cohort of immunomodulation therapy, there was high correlation between PD-1 and CTLA-4, CD25 and PD-1, followed by Foxp3; CD28 and Foxp3, TGF-β and IL-10, followed by Foxp3. There was reverse correlation (20%-40% cases) between PD-1 and TGF-β, CTLA-4 and TGF-β, CD25 and TGF-β, CD28 and TGF-β, followed by IL-10; Foxp3 and CTLA-4 followed by IL-10; IL-10 and CTLA-4. However, in the cohort with radiotherapy, the increase of PD-1 mRNA expression was companied by all six genes, but there was only minimal reverse correlation(<15% cases) between CTLA-4 and CD25, CD28 and IL-10, Foxp3 and CD28, TGF-β and CD28, IL-10 and CD28. When contrasting the mRNA levels of PD-1 and CTLA-4 between radiotherapy and immunomodulation therapy, it was found that they were mostly up-modulated after immunomodulation therapy and down-modulated after radiotherapy. These results indicate that the two types of therapy mediate immune responses through different immunoregulatory mechanisms, and that the immunomodulation therapy has much higher immunoregulatory activity. We did not observe correlation between PD-1 and Foxp3, though PD-1 and CTLA-4 up-modulation was often associated with CD25 up-regulation, suggesting their immunoactivation-derived property, without regulatory T cell induction [29, 31]. In addition, in comparison with immunomodulation therapy, radiation therapy was associated with increased TGF-β expression. The induced TGF-β might be immune-cell-derived or radiation-damage-induced [46–48], but regardless of its origin, the increased TGF-β expression likely affects the antitumor immunity, thus indicating the necessity of further immunomodulation therapy immediately after radiation therapy for some patients. In future, we will investigate the effect of such immunomodulation on antitumor immunity and function of the PD-1/PD-L1 signaling pathway.

In summary, our results demonstrate modulation of peripheral blood PD-1 mRNA levels after antitumor treatment. Our data show that the PD-1 gene expression is increased in peripheral blood in cancer patients, and can be modulated by clinical interventions. Cytokine immunomodulation therapy and ionizing irradiation have different immunomodulation effect; the immunomodulation therapy is associated with higher immunoreactivity and cytokine-induced PD-1 expression. Moreover, the PD-1 expression on peripheral lymphocytes can be immunoactivation-derived, different from that in TILs. Together, our data suggest that through combined analysis of PD-1, CTLA-4, CD25, CD28, Foxp3, TGF-ß and IL-10 in peripheral blood, and the lymphocyte subpopulations, we may accurately evaluate the functional status of cellular immunity, thus providing clues for guiding blockade of PD-1/PD-L1 signaling pathway for cancer treatment.

## MATERIALS AND METHODS

### Patients and blood sample preparation

Blood samples were obtained from patients with lung (n=27), renal (n=2), breast (n=5), gastrointestinal (n=4), and other malignancies (n=7) prior to or after radiotherapy and immunomodulation therapy with cytokines. Blood samples from healthy adults were freshly obtained from the blood bank in our hospital. Written consents were obtained from all patients. The protocol was approved by the Human Ethics Committee of the Affiliated Hospital of the Academy of Military Medical Sciences, Beijing, China, and was conducted in accordance with the Declaration of Helsinki. Whole blood (8 ml) was drawn into heparinized tubes for cytometry and RT-PCR analysis.

### Analysis of the cellular immunity

For monitoring the cellular immunity, T cells, B cells, NK cells, Treg cells, and lymphocyte subpopulations were analyzed by a 4-color flow cytometry (EPICS XL, Beckman Coulter Inc., USA). Fluorescein isothiocyanate (FITC)-, PE-Cy5-, PerCP-, allophycocyanin (APC)-, or PE-Texas Red (ECD)-conjugated antibodies against CD3, CD4, CD8, CD56, CD19, CD25, CD28, HLA-DR, CD45RA, and CD45RO were purchased from Beckman Coulter Inc. Cells were labeled according to the manufacturer's protocols.

### Analysis of gene mRNA expression

Expression of CD25, CD28, IL-10, TGF-β, Foxp3, CTLA-4, and PD-1 was analyzed by a real time RT-PCR (Beijing Mo Li Tai Bio-Technology Inc., Cat:201411). Briefly, lymphocytes were collected from peripheral blood of the patients by centrifugation at 400 × g for 5 minutes. The cells collected were then mixed with a red blood cell lysing solution (Bejing Dong Fang Hua Hui Biomedical Technology, Cat:21510) at 1:9 ratio and centrifuged Total RNA was isolated using a standard RNA isolation procedure according to the manufacturer (Life technologies, Cat: 15596-026). The RNA was then reversely transcribed into cDNA using a reverse transcription system (Promega, Cat: A5300). Real time PCR was performed using a 7500 real time PCR system (Applied Biosystems); β-actin was employed as the control gene.

### Immunomodulation therapy

Immunomodulation therapy with cytokines was designed according to the cellular immunity analyzed by flow cytometry [17]. Briefly, IFN-α-1b (Beijing Tri-Prime Gene Inc.), IL-2 (Beijing Shuanglu Phamacutical Inc. China) and Thymalfasin (Hainan Zhonghe Pharmaceutical CO. LTD) were used. The doses were 300 MU of IFN-α, 200 MU of IL-2 and 1mg of Thymalfasin for each injection. The modification was that, if the CD4/CD8 ratio was <1, 1.6 mg of Thymalfasin was added at every other day for four to six weeks. In addition, the following prerequisites were needed for the patients to receive the therapy: the absolute neutrophil count >0.3×10^9^/L and platelet count >70×10^9^/L, normal liver and renal function, at least two weeks after chemotherapy or operation, after wound healing, without gastrointestinal tract bleeding and without a history of allergy to cytokines. If over-induced Treg, down-regulated NK and abnormal CD4/CD8 ratio were not reversed (Treg/lymphocyte <3%, NK/lymphocyte >10%, CD4/CD8 ratio>1, another course was given.

### Radiotherapy

CyberKnifeand intensity modulated radiation therapy(IMRT) was employed for routine cancer radiotherapy. The total radiation doses were 32.5 to 37.5 GY for CyberKnife, divided into 5 fractions within seven days, and 60 to 70 GY for IMRT, divided into 30-35 fractions, 2GY per fraction, and 5 fractions per week.

### Statistical analysis

Statistical analyses of data were performed using GraphPad-Prism software (San Diego, CA). Calculations were performed as mean ± SEM and significance was denoted as *, p<0.05; **, p<0.01; and ***, p<0.001.

## SUPPLEMENTARY MATERIALS TABLES




