# Supplementary material for: PD-1 mRNA expression in peripheral blood cells and its modulation characteristics in cancer patients
Source: Oncotarget. 2017 Feb 2;8(31):50782–91. doi: 10.18632/oncotarget.15006 (PMC5584204; doi:10.18632/oncotarget.15006)
Supplement: Supplementary file 1 [file oncotarget-08-50782-s001.pdf]

PD-1 mRNA expression in peripheral blood cells and its modulation characteristics in cancer patients

SUPPLEMENTARY TABLES

Supplementary Table 1: Gene expression levels in peripheral blood of normal adults

	Relative gene expression [Absolute quantitative (target gene/housekeeping gene)]						
NO.	CD25	CD28	CTLA-4	PD-1	Foxp3	TGF-β	IL-10
#1	3.14E-04	6.69E-04	9.82E-04	6.03E-04	2.31E-02	7.08E-02	6.98E-02
#2	4.96E-04	6.90E-04	1.19E-03	9.86E-04	8.98E-03	6.60E-02	6.17E-02
#3	2.78E-04	6.12E-04	6.94E-04	3.45E-04	1.49E-03	7.83E-02	1.05E-01
#4	3.94E-04	5.72E-04	1.26E-03	4.31E-04	1.10E-03	8.72E-02	7.67E-02
#5	5.51E-04	5.82E-04	1.01E-03	2.59E-04	1.02E-03	7.51E-02	5.71E-02
#6	2.42E-04	5.59E-04	8.57E-04	2.02E-04	9.19E-04	1.07E-01	9.55E-02
#7	3.32E-03	2.02E-03	2.76E-03	1.26E-03	3.65E-03	9.78E-02	8.21E-02
#8	1.03E-03	8.00E-04	1.72E-03	3.08E-04	2.88E-03	6.74E-02	7.97E-02
#9	2.84E-03	7.86E-04	1.66E-03	3.04E-04	2.64E-03	6.24E-02	8.03E-02
#10	1.41E-04	2.64E-04	4.69E-04	5.99E-04	1.51E-03	6.03E-02	7.32E-02

Supplementary Table 2: Gene expression levels in peripheral blood of malignant patients

See Supplementary File 1

Supplementary Table 3: The proportion of T, NK, B, Treg, $CD3^+CD8^+CD28^+$ and $CD3^+$ $CD8^+CD28^-$ subpopulations in peripheral blood of malignant patients

[T cells: $CD3^+CD19^-$; B cells: $CD3^-CD19^+$; NK cells: $CD3^-CD16^+56^+$; Treg cells: $CD4^+CD25^+CD127^{low}$; suppressor T cells (Ts): $CD3^+CD8^+CD28^-$; cytotoxic T cells (CTL): $CD3^+CD8^+CD28^+$.]

See Supplementary File 1
