# Supplementary material for: PD-1 mRNA expression in peripheral blood cells and its modulation characteristics in cancer patients
Source: Oncotarget. 2017 Feb 2;8(31):50782–91. doi: 10.18632/oncotarget.15006 (PMC5584204; doi:10.18632/oncotarget.15006)
Supplement: Supplementary file 2 [file oncotarget-08-50782-s002.docx]

**PD-1 mRNA expression in peripheral blood cells and its modulation characteristics in cancer patients**

**Supplemental Tables:**

**Supplementary Table 2. Gene expression levels in peripheral blood of** **malignant patients**

(Patients #1-#25: immunomodulation therapy; Patients #26-#45: radiotherapy)

|  | **Relative gene expression**  **[Absolute quantitative (target gene/housekeeping gene)]** | | | | | | |
| --- | --- | --- | --- | --- | --- | --- | --- |
| No. | CD25 | CD28 | CTLA-4 | PD-1 | Foxp3 | TGF-β | IL-10 |
| #1 | 4.54E-01 | 7.37E-01 | 7.38E-01 | 1.46E-01 | 3.11E-01 | 3.87E+00 | 4.73E-01 |
| #2 | 3.03E-01 | 5.70E-01 | 1.32E-01 | 7.50E-02 | 1.26E+00 | 1.40E+01 | 2.38E+01 |
| #3 | 1.15E+00 | 3.40E-01 | 8.87E-02 | 3.19E-02 | 2.10E-02 | 1.62E+01 | 2.42E-01 |
| #4 | 5.37E-01 | 5.88E-01 | 9.42E-01 | 2.90E-01 | 5.57E-02 | 9.05E-01 | 5.29E-01 |
| #5 | 1.37E-04 | 5.62E-05 | 2.57E-04 | 1.96E-06 | 7.79E-06 | 5.90E-04 | 7.66E-05 |
| #6 | 8.20E-04 | 4.18E-03 | 5.64E-03 | 3.94E-04 | 4.35E-03 | 3.53E-02 | 8.06E-02 |
| #7 | 3.67E-01 | 3.77E-01 | 1.17E-01 | 7.60E-02 | 3.66E-01 | 1.07E+01 | 1.68E+01 |
| #8 | 2.40E-03 | 2.82E-03 | 8.50E-03 | 5.52E-04 | 1.65E-03 | 2.56E-02 | 1.87E-03 |
| #9 | 9.31E-01 | 7.95E-01 | 1.11E+00 | 7.72E-01 | 5.76E-02 | 5.33E-01 | 3.23E-01 |
| #10 | 1.42E-02 | 8.59E-02 | 2.71E-02 | 1.43E-02 | 1.33E-01 | 2.67E+00 | 3.11E+00 |
| #11 | 7.37E-02 | 6.04E-01 | 4.23E-02 | 3.63E-01 | 2.11E-01 | 2.20E+00 | 6.43E+00 |
| #12 | 7.54E-01 | 3.66E-01 | 7.26E-01 | 1.28E-01 | 1.52E+00 | 5.44E+00 | 1.03E+01 |
| #13 | 5.90E-02 | 2.84E-01 | 2.68E-02 | 1.11E-02 | 4.79E-01 | 1.16E+01 | 1.62E+01 |
| #14 | 7.06E-01 | 6.26E-01 | 9.02E-01 | 2.65E-01 | 2.45E-01 | 1.22E-01 | 7.90E-01 |
| #15 | 1.04E+00 | 7.09E-01 | 1.04E+00 | 3.56E-01 | 3.16E-01 | 2.67E-01 | 5.96E-01 |
| #16 | 1.46E-01 | 6.12E-01 | 2.62E-01 | 4.24E-01 | 2.73E-01 | 1.52E+01 | 5.30E-01 |
| #17 | 9.42E-01 | 2.20E-01 | 6.09E-01 | 2.62E-01 | 2.48E-02 | 5.13E-02 | 5.15E-01 |
| #18 | 7.81E-01 | 1.29E+00 | 7.82E-01 | 8.07E-01 | 1.47E+00 | 1.36E-01 | 7.48E-01 |
| #19 | 3.49E-01 | 1.20E+00 | 9.46E-01 | 6.38E-01 | 1.94E+00 | 8.27E-02 | 3.09E-01 |
| #20 | 7.94E-01 | 9.24E-01 | 1.10E+00 | 7.87E-01 | 5.51E-01 | 2.75E-01 | 7.93E-01 |
| #21 | 1.44E+00 | 7.62E-04 | 3.66E-01 | 1.38E-01 | 1.44E+00 | 1.61E+01 | 2.35E+01 |
| #22 | 7.88E-03 | 1.05E-02 | 3.72E-03 | 2.53E-05 | 1.57E-03 | 3.88E-02 | 4.46E-03 |
| #23 | 6.42E-02 | 1.96E-02 | 1.02E-02 | 1.30E-03 | 1.95E-03 | 2.99E-01 | 5.28E-03 |
| #24 | 1.60E-01 | 7.02E-01 | 6.42E-01 | 3.09E-01 | 7.63E-01 | 5.05E-02 | 1.31E-01 |
| #25 | 4.31E-01 | 9.33E-02 | 3.21E-01 | 4.38E-02 | 7.03E-02 | 1.27E-01 | 1.88E-01 |
| #26 | 4.66E-01 | 1.70E-01 | 4.30E-01 | 4.00E-02 | 3.38E-03 | 4.11E+01 | 1.94E+00 |
| #27 | 7.54E-01 | 1.30E-01 | 4.14E-01 | 5.37E-02 | 6.57E-03 | 3.51E+01 | 8.89E-01 |
| #28 | 1.15E+00 | 3.80E-01 | 8.02E-01 | 1.44E-02 | 3.24E-03 | 1.76E+01 | 3.17E+00 |
| #29 | 2.84E-01 | 1.35E-01 | 2.58E-01 | 2.79E-02 | 4.47E-01 | 1.06E+00 | 8.18E+00 |
| #30 | 5.33E+01 | 6.38E+00 | 4.42E+00 | 8.25E-01 | 3.31E-01 | 1.38E-02 | 9.87E+00 |
| #31 | 1.65E+00 | 4.58E-01 | 2.70E-01 | 1.56E-01 | 2.16E-02 | 2.60E+00 | 1.63E+00 |
| #32 | 3.54E-01 | 4.82E-02 | 1.30E+00 | 3.67E-02 | 2.30E-03 | 1.90E-01 | 4.03E-01 |
| #33 | 9.49E-01 | 2.34E-01 | 4.44E-01 | 1.21E-01 | 4.02E-01 | 1.15E-01 | 8.86E-02 |
| #34 | 1.31E-03 | 2.29E-03 | 4.70E-02 | 8.51E-04 | 1.29E-03 | 1.44E-02 | 3.75E-02 |
| #35 | 1.35E-03 | 1.09E-03 | 1.80E+00 | 1.80E-04 | 3.77E-03 | 3.50E-03 | 1.72E-02 |
| #36 | 2.09E-03 | 3.49E-04 | 5.61E-04 | 7.79E-05 | 1.13E-04 | 3.39E-03 | 5.06E-04 |
| #37 | 2.38E-03 | 2.08E-03 | 3.32E-02 | 1.90E-05 | 4.27E-04 | 1.68E-03 | 4.57E-03 |
| #38 | 1.48E-02 | 2.93E-03 | 7.92E-03 | 4.44E-05 | 1.67E-03 | 1.04E-02 | 5.71E-03 |
| #39 | 2.11E-03 | 1.04E-02 | 1.28E-03 | 3.47E-06 | 1.25E-03 | 1.96E-03 | 9.47E-03 |
| #40 | 7.27E-01 | 2.04E-01 | 5.60E-01 | 4.23E-01 | 5.89E-02 | 2.35E-02 | 3.72E-01 |
| #41 | 6.93E-04 | 6.07E-03 | 2.12E-03 | 7.81E-03 | 6.78E-02 | 1.55E-01 | 1.33E-01 |
| #42 | 1.02E-03 | 3.32E-04 | 4.19E-04 | 1.29E-05 | 5.38E-05 | 2.79E-03 | 1.93E-04 |
| #43 | 7.18E-04 | 2.00E-02 | 5.50E-03 | 5.63E-05 | 3.41E-04 | 1.77E-03 | 3.30E-03 |
| #44 | 3.28E-01 | 5.59E-02 | 6.09E-01 | 1.01E-01 | 2.14E-02 | 9.77E-02 | 2.09E-01 |
| #45 | 4.71E-03 | 1.61E-03 | 1.66E-03 | 2.53E-05 | 3.01E-04 | 1.29E-02 | 6.97E-04 |

**Supplementary Table 3. The proportion of T, NK, B, Treg, CD3^+^CD8^+^CD28^+^ and CD3^+^ CD8^+^CD28^-^ subpopulations in peripheral blood of malignant patients**

**[T cells:** CD3^+^CD19^-^; **B cells:** CD3^-^CD19^+^; **NK cells:** CD3^-^CD16^+^56^+^; **Treg cells:** CD4^+^CD25^+^CD127^low^; **suppressor T cells (Ts):** CD3^+^CD8^+^CD28^-^; **cytotoxic T cells (CTL):** CD3^+^CD8^+^CD28^+^.]

| **No.** | **Date(d/m/y)** | **T** | **B** | **NK** | **Treg** | **Ts** | **CTL** |
| --- | --- | --- | --- | --- | --- | --- | --- |
| #1 | 5/6/15 | 72.10% | 10.10% | 17.30% | 4.11% | 1.83% | 26.70% |
|  | 12/6/15 | 70.20% | 7.50% | 23.00% | 5.28% | 2.14% | 22.80% |
| #2 | 28/11/14 | 64.50% | 1.20% | 31.40% | 2.37% | 31.60% | 7.53% |
|  | 16/12/16 | 69.6% | 1.68% | 30.7% | 2.32% | 30.3% | 8.48% |
| #3 | 3/11/15 | 67.0% | 24.9% | 5.46% | 3.78% | 11.9% | 21.8% |
|  | 12/11/15 | 71.6% | 22.9% | 6.66% | 5.08% | 13.4% | 20.7% |
| #4 | 12/6/15 | 72.80% | 7.57% | 20.00% | 1.71% | 20.90% | 11.00% |
|  | 19/6/15 | 73.00% | 6.95% | 20.10% | 3.33% | 17.70% | 9.56% |
| #5 | 9/9/15 | 78.70% | 4.38% | 16.70% | 2.55% | 33.10% | 6.39% |
|  | 16/9/15 | 76.60% | 5.33% | 16.40% | 1.31% | 32.20% | 6.32% |
| #6 | 27/1/15 | 80.60% | 5.27% | 14.90% | 3.45% | 13.40% | 20.50% |
|  | 3/2/15 | 76.10% | 4.32% | 18.30% | 4.05% | 12.80% | 20.60% |
| #7 | 26/11/14 | 75.80% | 1.64% | 19.10% | 3.92% | 45.10% | 9.61% |
|  | 20/3/15 | 71.40% | 7.76% | 20.30% | 1.64% | 42.20% | 6.34% |
| #8 | 19/6/15 | 46.00% | 12.40% | 41.90% | 1.63% | 11.20% | 9.12% |
|  | 26/6/15 | 35.40% | 10.50% | 55.10% | 2.06% | 9.32% | 6.02% |
| #9 | 5/6/15 | 77.50% | 20.30% | 6.37% | 3.92% | 9.25% | 12.80% |
|  | 12/6/15 | 80.20% | 11.10% | 8.69% | 4.62% | 11.30% | 13.90% |
| #10 | 5/3/15 | 65.10% | 19.00% | 15.40% | 3.91% | 2.78% | 15.80% |
|  | 12/3/15 | 67.60% | 15.20% | 16.80% | 3.25% | 3.53% | 15.50% |
| #11 | 25/11/14 | 78.30% | 9.36% | 12.60% | 2.68% | 23.30% | 15.50% |
|  | 12/6/15 | 77.4% | 13.5% | 9.48% | 2.20% | 20.5% | 16.6% |
| #12 | 14/11/14 | 84.40% | 6.66% | 9.51% | 2.94% | 41.70% | 9.12% |
|  | 17/3/15 | 83.50% | 7.48% | 8.52% | 2.41% | 38.50% | 10.80% |
| #13 | 15-3-9 | 75.00% | 19.50% | 5.31% | 3.26% | 0.68% | 19.20% |
|  | 24/9/15 | 74.60% | 16.50% | 8.18% | 3.85% | 2.02% | 18.50% |
| #14 | 12/5/15 | 82.60% | 6.08% | 10.90% | 6.89% | 5.31% | 19.70% |
|  | 28/5/15 | 80.80% | 3.48% | 15.00% | 7.38% | 5.57% | 18.50% |
| #15 | 13/5/15 | 66.30% | 6.70% | 46.60% | 2.95% | 12.70% | 20.80% |
|  | 19/5/15 | 64.00% | 10.90% | 25.30% | 4.72% | 6.31% | 19.70% |
| #16 | 3/4/15 | 60.00% | 2.38% | 36.00% | 5.25% | 9.82% | 13.20% |
|  | 10/4/15 | 57.30% | 3.59% | 38.10% | 4.58% | 11.60% | 13.30% |
| #17 | 17/9/15 | 54.90% | 21.20% | 23.00% | 1.26% | 10.10% | 14.80% |
|  | 23/9/15 | 50.10% | 28.70% | 22.50% | 1.50% | 8.94% | 12.50% |
| #18 | 22/12/14 | 75.80% | 13.90% | 9.65% | 2.82% | 9.09% | 14.70% |
|  | 12/5/15 | 73.10% | 15.80% | 15.80% | 3.01% | 9.03% | 11.90% |
| #19 | 13/3/15 | 64.90% | 21.60% | 14.20% | 3.91% | 15.00% | 16.20% |
|  | 20/3/15 | 67.40% | 18.00% | 14.40% | 3.14% | 16.10% | 18.90% |
| #20 | 13/5/15 | 93.80% | 0.50% | 7.45% | 3.40% | 18.30% | 22.60% |
|  | 19/5/15 | 75.80% | 1.41% | 23.90% | 6.26% | 14.60% | 17.00% |
| #21 | 25/11/14 | 63.50% | 9.39% | 26.50% | 1.75% | 16.20% | 15.60% |
|  | 1/12/14 | 72.20% | 7.25% | 20.20% | 4.01% | 24.20% | 17.20% |
| #22 | 9/7/15 | 72.30% | 16.20% | 10.70% | 3.07% | 11.80% | 24.10% |
|  | 14/7/15 | 73.00% | 13.90% | 13.90% | 4.31% | 11.40% | 23.10% |
| #23 | 30/10/15 | 78.10% | 15.80% | 6.58% | 4.32% | 16.10% | 19.70% |
|  | 4/11/15 | 80.10% | 13.30% | 6.72% | 3.53% | 15.60% | 20.40% |
| #24 | 16/3/15 | 51.70% | 21.70% | 25.60% | 4.99% | 3.20% | 7.60% |
|  | 23/3/15 | 55.40% | 25.20% | 17.80% | 5.58% | 3.37% | 8.12% |
| #25 | 21/7/15 | 88.40% | 3.92% | 7.35% | 4.67% | 13.20% | 17.80% |
|  | 28/7/15 | 78.60% | 4.24% | 16.30% | 3.09% | 20.80% | 15.10% |
| #26 | 24/9/15 | 65.40% | 16.50% | 17.40% | 3.26% | 12.70% | 16.40% |
|  | 17/11/15 | 80.50% | 9.21% | 8.97% | 4.61% | 9.34% | 27.10% |
| #27 | 2/9/15 | 77.00% | 5.84% | 17.00% | 3.48% | 13.50% | 17.20% |
|  | 9/9/15 | 62.90% | 3.35% | 34.80% | 3.53% | 17.30% | 11.20% |
| #28 | 31/8/15 | 62.80% | 19.00% | 17.30% | 4.90% | 5.03% | 11.00% |
|  | 26/10/15 | 61.70% | 5.15% | 32.10% | 5.94% | 14.40% | 12.10% |
| #29 | 6/11/15 | 76.80% | 4.87% | 17.00% | 2.67% | 10.80% | 19.30% |
|  | 8/1/16 | 55.30% | 0.89% | 41.40% | 1.31% | 11.80% | 13.30% |
| #30 | 7/9/15 | 80.10% | 9.38% | 8.62% | 3.37% | 31.70% | 11.80% |
|  | 16/10/15 | 79.9% | 12.8% | 5.54% | 4.19% | 30.4% | 10.6% |
| #31 | 14/10/15 | 58.00% | 12.70% | 16.80% | 2.42% | 2.41% | 23.10% |
|  | 24/12/15 | 79.40% | 1.39% | 17.30% | 2.01% | 26.90% | 16.90% |
| #32 | 31/7/15 | 68.40% | 7.32% | 25.80% | 2.40% | 26.80% | 12.50% |
|  | 11/9/15 | 72.10% | 2.66% | 24.70% | 2.21% | 28.20% | 13.30% |
| #33 | 10/10/15 | 74.30% | 3.01% | 23.00% | 2.86% | 21.60% | 16.20% |
|  | 10/12/15 | 85.70% | 1.41% | 13.90% | 3.62% | 27.40% | 16.40% |
| #34 | 24/11/15 | 84.50% | 2.63% | 12.00% | 1.73% | 38.80% | 6.08% |
|  | 29/12/15 | 77.10% | 0.79% | 16.80% | 2.15% | 33.00% | 13.80% |
| #35 | 7/12/15 | 77.80% | 5.60% | 16.30% | 3.43% | 20.80% | 11.00% |
|  | 25/12/15 | 68.80% | 0.42% | 29.70% | 2.51% | 19.00% | 6.53% |
| #36 | 9/9/15 | 65.40% | 16.50% | 17.40% | 3.26% | 12.70% | 16.40% |
|  | 13/10/15 | 80.50% | 9.21% | 8.97% | 4.61% | 9.34% | 27.10% |
| #37 | 24/11/15 | 76.30% | 9.35% | 12.50% | 3.01% | 16.10% | 14.30% |
|  | 20/1/15 | 85.40% | 5.23% | 7.89% | 3.41% | 14.60% | 13.50% |
| #38 | 8/7/15 | 77.00% | 8.00% | 15.30% | 6.27% | 6.88% | 18.10% |
|  | 8/21/15 | 90.2% | 2.84% | 6.12% | 6.65% | 12.6% | 19.7% |
| #39 | 9/11/15 | 42.40% | 2.94% | 53.60% | 1.72% | 10.20% | 12.20% |
|  | 22/12/15 | 47.60% | 1.38% | 47.90% | 2.25% | 10.30% | 12.40% |
| #40 | 25/9/15 | 66.40% | 11.40% | 26.00% | 1.59% | 9.89% | 12.10% |
|  | 16/10/15 | 63.90% | 4.12% | 29.70% | 2.51% | 12.70% | 12.60% |
| #41 | 5/1/16 | 70.00% | 15.90% | 14.10% | 3.61% | 6.64% | 7.62% |
|  | 14/1/16 | 75.30% | 5.83% | 20.40% | 4.48% | 7.20% | 6.98% |
| #42 | 14/9/15 | 78.50% | 9.64% | 12.30% | 2.12% | 14.20% | 20.40% |
|  | 28/10/15 | 89.80% | 2.44% | 5.56% | 4.02% | 10.00% | 23.40% |
| #43 | 2/11/15 | 76.60% | 2.40% | 22.40% | 2.63% | 21.80% | 9.44% |
|  | 21/12/15 | 77.30% | 1.06% | 22.40% | 1.87% | 26.00% | 9.56% |
| #44 | 23/7/15 | 50.10% | 4.31% | 45.20% | 2.46% | 8.19% | 9.66% |
|  | 8/9/15 | 53.1% | 3.97% | 40.5% | 2.55% | 13.4% | 11.3% |
| #45 | 10/9/15 | 86.30% | 4.36% | 9.48% | 4.72% | 12.50% | 21.80% |
|  | 23/10/15 | 88.30% | 2.61% | 8.96% | 3.09% | 13.00% | 21.10% |
